# S6-1 Implementing interventions to promote physical activity among middle aged and older adults: opportunities and challenges

**DOI:** 10.1093/eurpub/ckad133.028

**Published:** 2023-09-11

**Authors:** Chair(s):, Janet Boekhout, Denise Peels

**Affiliations:** Open Universiteit; Open Universiteit, The Netherlands

## Abstract

Extensive research has demonstrated the effectiveness of physical activity interventions for middle aged and older adults (MAOA). Despite this effectiveness, the actual reach of the target population participating in these interventions in a real-world setting remains relatively low. This demonstrates the urgent need for further implementation research. In the Netherlands, this need has been recognized earlier, leading to the foundation of the ZonMW financed Implementation Network Sports and Physical Activity: the presenters in this symposium are all members of that network.

The aim of this symposium is to describe barriers and facilitators regarding implementation that were determined by qualitative studies among MOAO and stakeholders. In addition, the development of a practical tool that stimulates implementation by stakeholders is presented. These studies were grounded in relevant scientific frameworks such as Consolidated Framework for Implementation Research (CFIR) and Implementation Mapping. This symposium also provides recommendations for future research and practice in the shape of potential strategies that can be applied when implementing interventions.

